# Biomimetic nanoparticles for tumor immunotherapy

**DOI:** 10.3389/fbioe.2022.989881

**Published:** 2022-11-09

**Authors:** Hanqing Yu, Meng Wu, Siyu Chen, Mingming Song, Yulin Yue

**Affiliations:** ^1^ Department of Clinical Laboratory, Children’s Hospital of Nanjing Medical University, Nanjing, China; ^2^ School of Life Science and Technology, China Pharmaceutical University, Nanjing, China

**Keywords:** tumor microenvironment, cell membrane, biomimetic nanoparticles, immunotherapy, nano drug delivery systems

## Abstract

Currently, tumor treatment research still focuses on the cancer cells themselves, but the fact that the immune system plays an important role in inhibiting tumor development cannot be ignored. The activation of the immune system depends on the difference between self and non-self. Unfortunately, cancer is characterized by genetic changes in the host cells that lead to uncontrolled cell proliferation and evade immune surveillance. Cancer immunotherapy aims to coordinate a patient’s immune system to target, fight, and destroy cancer cells without destroying the normal cells. Nevertheless, antitumor immunity driven by the autoimmune system alone may be inadequate for treatment. The development of drug delivery systems (DDS) based on nanoparticles can not only promote immunotherapy but also improve the immunosuppressive tumor microenvironment (ITM), which provides promising strategies for cancer treatment. However, conventional nano drug delivery systems (NDDS) are subject to several limitations in clinical transformation, such as immunogenicity and the potential toxicity risks of the carrier materials, premature drug leakage at off-target sites during circulation and drug load content. In order to address these limitations, this paper reviews the trends and progress of biomimetic NDDS and discusses the applications of each biomimetic system in tumor immunotherapy. Furthermore, we review the various combination immunotherapies based on biomimetic NDDS and key considerations for clinical transformation.

## 1 Introduction

Cancer is a major public health problem worldwide and the second leading cause of death in the world (Castle et al., 2021; Soerjomataram and Bray, 2021; Xi and Xu, 2021). An estimated 4 million newly diagnosed cancer cases and 3 million cancer deaths occurred in China in 2020 (Soerjomataram and Bray, 2021; Xia et al., 2022). Cancer is being treated with conventional therapies, including surgery, chemotherapy, and localized radiation therapy (Chhatre et al., 2021; Najafi et al., 2021; Wang and Tepper, 2021). However, cancer metastasis and recurrence lead to treatment failure (Ganesh and Massague, 2021). Cancer is a systemic disease, and long-term inflammation is a hallmark of cancer (Haskins et al., 2020; Markus et al., 2021). Whether this inflammation triggers or supports tumor growth depends on the immune environment (Ganesh and Massague, 2021).

The advent of immunotherapy has changed the paradigm of cancer treatment (Rey-Cardenas et al., 2021). Immunotherapy mainly improves the antitumor immune response and produces fewer off-target effects than chemotherapy and other drugs that directly kill the cancer cells (Zeng and Pu, 2020a; Esfahani et al., 2020; Duan and Luo, 2021). In immunotherapy, drugs are used directly to activate or promote the activation of the immune system to attack the cancer cells through natural mechanisms (Riley et al., 2019; Mortezaee and Najafi, 2021). Immune cells are involved in or associated with the immune response, and mainly include lymphocytes, mononuclear macrophages, dendritic cells, granulocytes, hematopoietic stem cells, etc., (Conceicao-Silva et al., 2021). Depending on their functional, immune cells are characterized as antigen-specific lymphocytes, antigen-presenting cells and other cells involved in the immune response (Shao et al., 2018). Antigen-specific lymphocytes, including T and B lymphocytes, play a central role in the immune response (Chen and Shen, 2020). The main function of T cells is to mediate adaptive cellular immunity, and they differentiate into effector Th1 cells and effector T cells. At the meanwhile, T cells facilitate the auxiliary adaptive humoral immunity, differentiating into the helper T cell 2 and helper B cell. Furthermore, the helper B cell will further activate, proliferate, differentiate to produce the antibody (Nandi et al., 2020). In addition, antigen-presenting cells include mononuclear macrophages, dendritic cells, natural killer cells, and granulocytes. Mononuclear macrophages are mainly responsible for phagocytosis, while releasing lysosomal enzymes, TNF-α and other factors to kill tumor cells and virus-infected cells (Peng and Fadeel, 2022). In addition to antigen presentation, dendritic cells also have the function of inducing T cell differentiation, establishing self-tolerance, regulating immunity and maintaining immune memory. Natural killer cells could counteract the infection, while possess antitumor effects by directly contacting with tumor cells or by binding specific IgG of tumor cells based on the antibody-dependent and cell-mediated cytotoxicity (ADCC) effects (Gauthier et al., 2021). Therefore, immunotherapy is considered a promising strategy for treating or curing certain types of cancer. In addition, chimeric antigen receptor T-cell immunotherapy (CAR-T therapy) has been shown to be effective in leukemia patients (Feins et al., 2019; Zhang et al., 2020a). For example, in 2010, sipuleucel-T was approved for the treatment of prostate cancer (Cheever and Higano, 2011). In 2011, the checkpoint inhibitor ipilimumab, a monoclonal antibody (mAb) targeting cytotoxic T lymphocyte antigen 4 (CTLA4), was approved for use with in advanced melanoma (Mullard, 2013). In addition, modulation of the immune system in existing patients by immune checkpoint inhibitors, such as anti-CTLA4, anti-PD-1, and anti-PDL-1 and CAR-T therapy have been developed and approved for clinical use (Yue et al., 2019; Waldman et al., 2020). These immunotherapies fall into several categories including checkpoint inhibitors, lymphocyte-activating cytokines, CAR-T cells, and other cell therapies (Riley et al., 2019). However, the clinical efficacy of immunotherapy for solid tumors is not satisfactory. The main reason is the difficulties in cell therapy of solid tumors, such as the heterogeneity of different solid tumor types, the lack of unique tumor-associated antigens as CAR-T targets, the inability of T cells to effectively localize to tumor sites, the insufficient persistence of CAR-T cells, and the complicated microenvironment in tumors that have inhibitory effects on immunity (Leko and Rosenberg, 2020; Morotti et al., 2021).

In addition, most cellular immunotherapies have been used primarily in patients with advanced cancer; so, response rates for the less advanced disease have yet to be fully determined (Sangro et al., 2021). However, existing cancer immunotherapies are inadequate because cancer antigens are not usually efficiently delivered to immune cells (Liang et al., 2021). Unlike lymphoma, solid tumors evade anticancer immunity by forming an tumor microenvironment (TME) (Park et al., 2018; Giraldo et al., 2019; Montironi et al., 2021). An established tumor is a complex tissue composed of not only tumor cells, but also stromal cells, inflammatory cells, vasculature, and extracellular matrix (ECM), all of which collectively are defined as TME (Foray et al., 2020). TME is where immune suppression and immune enhancement meet, but usually immune suppression dominates. Successful tumor control by immunotherapy requires activation of the immune system, expansion of effector cells, infiltration of activated effector cells into tumor tissue, and destruction of tumor cells (Wei et al., 2020). However, TME often hinders the sensitization of effector lymphocytes, reduces their infiltrating ability, and inhibits infiltrating effector cells, thus leading to the impairment of the antitumor effect of the body. Therefore, the further development of more extensive and effective immunotherapy strategies requires a deeper understanding of the immune relationship between tumors and their hosts (Sharma and Allison, 2015; Daver et al., 2021). There are several strategies that enable tumor cells to evade an immune response, and it is encouraging that some of these strategies have been successfully targeted to produce a durable antitumor immune response. First, cancer antigens must be efficiently transmitted to immune cells, especially antigen-presenting cells (APCs) (Liu et al., 2021a; Su et al., 2022). Second, inhibitory signaling pathways (called immune “checkpoints”) (Alemohammad et al., 2022) that limit the activation of immune cells can be suppressed, and this approach has been shown to induce the regression of melanoma and other types of cancer (Naimi et al., 2022). Finally, immunogenic cell death (ICD) inducers that initiate antitumor immune responses have shown great potential to reverse the ITM into immunoreactive tumors (Kepp et al., 2011; Jiang et al., 2021a). However, a key challenge in cancer immunotherapy remains the controllable regulation of the immune system, as these treatments suffer from severe adverse effects, including autoimmunity and nonspecific inflammation (Kolb et al., 2018; Phuengkham et al., 2019; Li et al., 2021a). Understanding how to improve the response efficiency of various immunotherapies is key to improving efficacy and managing these adverse effects. Currently, NDDS have demonstrated benefits in cancer treatment and management through good pharmacokinetics, precise targeting, and reduced side effects and drug resistance (Kumar et al., 2021; Xie et al., 2021).

**FIGURE 1:**
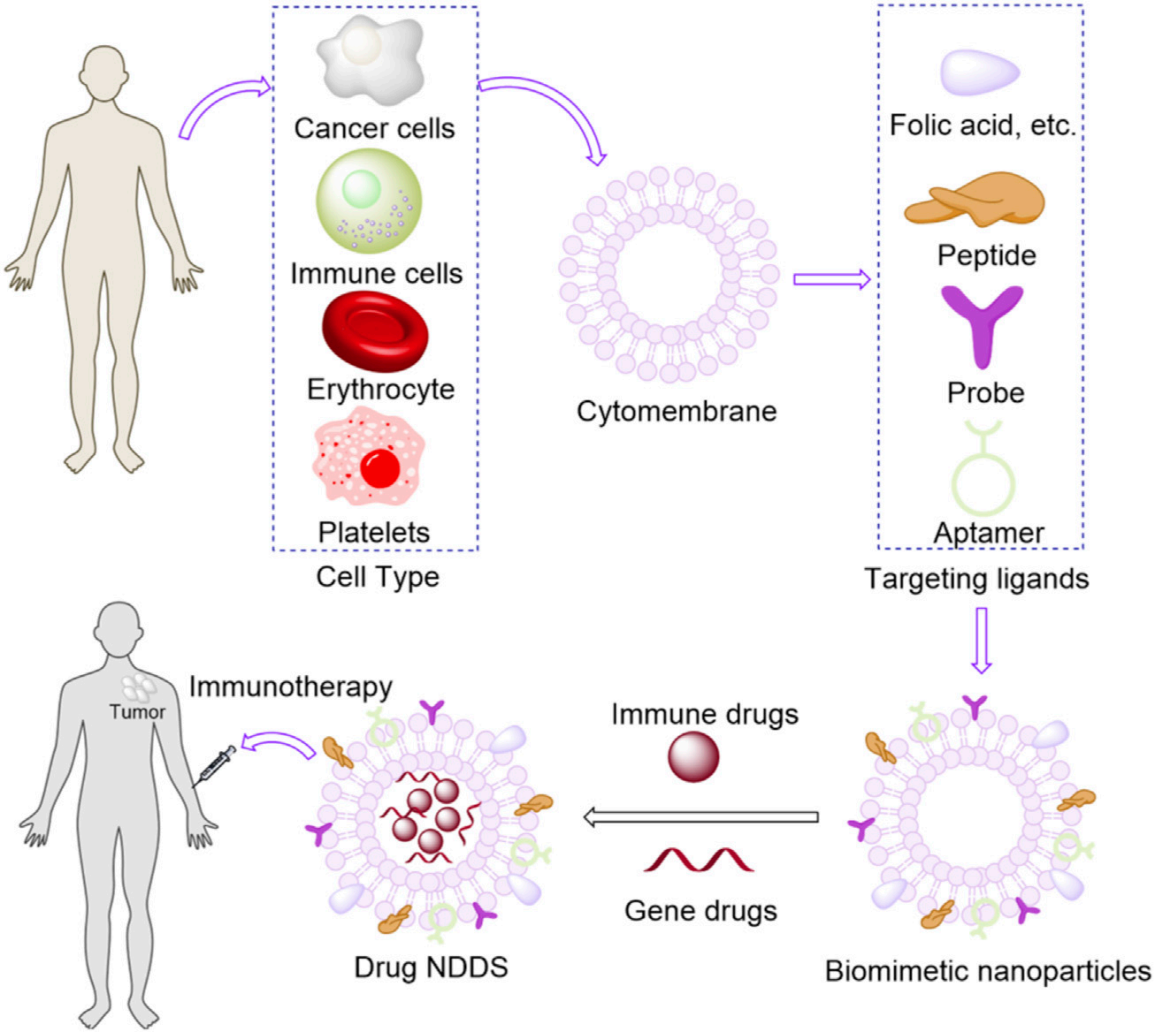
Biomimetic nanoparticles for tumor immunotherapy.

To address the limitations of current cancer treatments, this article reviews cancer immunotherapy strategies based on biomimetic nanodrug delivery systems (BNDDS), ranging from antigen/adjuvant delivery vehicles to compounds and their combinations targeting tumor antigen-specific T lymphocytes. In recent years, BNDDS have attracted great attention due to their potential applications in drug delivery Figure 1. Bionics refers to the extraction, isolation and purification of endogenous substances directly from humans, animals or microorganisms, or the synthesis of products similarly with structure and function to endogenous substances. The ultimate goal of these strategies is to reproduce the natural action pathways of biological structures in living organisms (Li et al., 2021b). These DDS can effectively improve the efficacy of immunotherapy and reduce toxic and side effects. In addition, we also discuss these advances, as well as the opportunities and challenges of integrating drug delivery technologies into cancer immunotherapy, and critically analyze the prospects of these emerging fields. Although bionic nanomedicine delivery technology has shown promising results in the field of immunization, the technology is still in its infancy. So far, these nanoscale therapies are still in the laboratory (Liu et al., 2021b). Based on the above-mentioned biomimetic materials from different sources, we summarize several key technical challenges in the preparation of these biomimetic nanomedicine delivery. First, membrane modification technology is not mature. The complexity and low reproducibility of the preparation process limit the scale of the preparation (Zhang et al., 2021a). Second, although a variety of modification strategies have been developed, reaction parameters, such as the conditions of preparation and the intrinsic environment, vary in different batches of experiments, lacking a necessary criterion. When modifying cell membranes, we must recognize that they are part of living entities (Zaman et al., 2021). In functionalization, appropriate reaction conditions should be controlled to avoid damaging cell activity. However, there is no judgment basis to select these conditions to improve the modification efficiency. Third, despite of the evaluation of membrane potential, the judging method to confirm the successful membrane modification is very limited (Caglar et al., 2019). In addition, Forth, particle size detection and morphology observation of the small modified molecules are difficult to be distinguished. Western blot analysis can only distinguish between the composition of modified and original membranes. Fifth, it is whether the activity of cell membrane is difficult to assess after modification (Wang et al., 2022a). Although gold nanoparticle antibody labeling can identify the direction of membrane coating, the process is complicated. It is of great urgent to design a new approach to visualize the membrane modification process (Akcapinar et al., 2021). Finally, the stability of the membrane is difficult to ensure in this engineering process. Predictably, the modification process interferes with surface activity and blocks membrane proteins. During prolonged reaction and preservation, viral and pyrogen contaminations are unavoidable, and membrane proteins often become denatured by the underlying immune response. As shown in Table 1, we summarize several typical classes of immunotherapies based on BNDDS.

**TABLE 1:** Summary of cell membrane-based biomimetic nanoparticles for cancer immunotherapy.

Types of cell membrane	Core material	Drugs	Applications	REF
Biomacromolecule	—	Paclitaxel and GANT61	Treatment against metastatic breast cancer	Jiang et al. (2021b)
		Lenvatinib and vadimezan	Cancer immunotherapy	Zheng et al. (2022)
		Withanolides	Treatment of triple negative breast cancer	Wang et al. (2019)
Cancer cell membrane	PLGA nanoparticles	R837 (an agonist against toll-like receptor 7)	Cancer immunotherapy	Yang et al. (2018)
		Indocyanine green (ICG) and decitabine (DCT)	Cancer immunotherapy	Zhao et al. (2020a)
	Mesoporous silica nanoparticles	Doxorubicin	Treatment of prostate cancer	Liu et al. (2019)
		Glucose oxidase	Cancer immunotherapy	Xie et al. (2019)
Immune cell membrane	Gelatin nanoparticles	Cancer cisplatin (Pt)	Head and neck squamous cell carcinoma	Rao et al. (2019)
	Fe_3_O_4_@SiO_2_ nanoparticles	Tumor-specific antigens	Cancer immunotherapy	Wu et al. (2021)
	mPEG-PLGA nanoparticles	4,4′,4″,4″-(porphine-5,10,15,20-tetrayl) tetrakis (benzoic acid)	Tumor immunotherapy	Deng et al. (2018)
	Lipid nanoparticles	Human interleukin (IL)-15 super-agonist	Enhancing T cell therapy	Tang et al. (2018)
	PLGA nanoparticles	Trametinib	Treatment of melanoma skin cancer	Yaman et al. (2020)
Platelet membrane	Fe_3_O_4_ nanoparticles	Sulfasalazine	Cancer immunotherapy	Jiang et al. (2020)
	PLA nanoparticles	Resiquimod (R848)	Enhances antitumor immunity	Bahmani et al. (2021)
	Silica particles	TRAIL	Cancer immunotherapy	Li et al. (2016)
Erythrocyte membrane	Quantum dot	Black phosphorus quantum dot (BPQDs)	Photothermal cancer immunotherapy	Liang et al. (2019)
	PLGA nanoparticles	Monophosphoryl lipid	Cancer immunotherapy	Guo et al. (2015)
	Ag nanoparticles	Anti–programmed death ligand 1 (PD-L1) blockade	Cancer immunotherapy	Han et al. (2019)

## 2 Mechanisms of cancer immunotherapy

A tumor is still considered as one of the most common and serious diseases, which directly damages human health (Wu et al., 2019; Britt et al., 2020). Compared with traditional chemotherapy (Ihrig et al., 2020), immunotherapy (Hegde and Chen, 2020) may induce a stronger sustained antitumor immune response in patients with advanced malignant tumors (Yu et al., 2019; Zhang et al., 2020b). Cancer immunotherapy is considered a treatment that aims to restore the immune system’s ability to recognize and reject cancer by removing tumor cells through the cancer–immune cycle (Carlberg and Velleuer, 2022; Finck et al., 2022; Lang et al., 2022; Tian et al., 2022). When cancer cells die through apoptosis or necrosis, tumor cell surface antigens are captured by APCs (eg, dendritic cells) (Zhang et al., 2022a; Zhang et al., 2022b). These dendritic cells, which carry cancer antigens, will migrate to the lymph nodes and provide the basis for stimulating T cell activation (Garris and Luke, 2020; Zhivaki et al., 2020; Kwiecien et al., 2021). These activated tumor-specific cytotoxic T lymphocytes will then infiltrate the tumor site and recognize tumor cells by interacting with T cell receptors (Cordell et al., 2022; De Sanctis et al., 2022). Ultimately, effector T cells kill cancer cells by inducing apoptosis, thereby releasing additional cancer antigens to further enhance the immune response (Liu and Sun, 2021). Currently, cancer immunotherapy techniques targeting different strategies have been developed, involving cancer vaccines (Guo et al., 2019), immune checkpoint block (ICB) (Tang et al., 2021), adoptive cell transfer (ACT) (Zhang et al., 2019), MAbs therapy (Francis et al., 2020), and cytokine therapy (Momin et al., 2019).

Cancer vaccines can be divided into cells, viral vectors, or molecules (peptides, DNA, or RNA) (Maiorano et al., 2021). Cellular vaccines are developed using autologous patient-derived tumor cells or cells derived from allogeneic tumor cell lines (Tomasicchio et al., 2019). Common cellular vaccines are divided into whole cell vaccines and dendritic cell vaccines (Wang et al., 2020a). A DNA or RNA vaccine is a gene that can be transfected and expressed in APC using genes encoding tumor antigens (Jahanafrooz et al., 2020). Physiologically, these antigens are further processed and presented to initiate a strong and viable antitumor immune response. The most important aspects of DNA vaccination are the selection or design of effective plasmid vectors and effective delivery systems and monitoring of the immune response after vaccination (Hobernik and Bros, 2018; Eusebio et al., 2021).

Immune checkpoint therapy is a cluster of therapies that modulate the activity of T cells to improve the immune response against tumors (Fritz and Lenardo, 2019). Currently, inhibition of immune checkpoint blocking signals to regulate T cell activity and enhance its anti-tumor effect is a hot topic in oncology therapy. For example, cytotoxic T lymphocyte antigen 4 (CTLA-4), PD-1, and PD-L1 are used ligand antagonists and other drugs interfere with immune checkpoints, which can directly stimulate the activation of cytotoxic T cells to initiate antitumor immunity and mediate sustained tumor suppression (Sadreddini et al., 2019).

Among these, CTLA-4 is highly expressed on the surface of activated T cells and binds to CD80 or CD86, inhibiting T cells from entering S phase from the G1 phase, and thus downregulating or terminating the T cell response (Gaynor et al., 2022). CTLA-4 produces a strong inhibitory signal that terminates T cell proliferation and activation of T cells. In addition, the PD-1 immunomodulatory system is likely with CTLA-4, but significant differences exist. Similarly, the CTLA-4 and PD-1 are expressed on the surface of TCR-activated T cells, but not on native and memory T cells. Unlike CTLA-4, PD-1 expression on the surface of activated T cells requires transcriptional activation, and therefore its expression is delayed by 6 h–12 h compared to CTLA-4 (Willsmore et al., 2021). However, immune checkpoint inhibitors still face challenges that need to be addressed. A major drawback of current immune checkpoint inhibitors is the lack of response to certain cancers, such as glioblastoma and pancreatic cancer, which may be attributed to their inherently low immunogenicity.

ACTs use a patient’s natural T cells, or natural killer cells, to detect and eliminate fast-growing tumors (Perrin et al., 2020). The first ACT was developed in 2002 (Dudley et al., 2002). These therapies have received a lot of attention in oncology over the past two decades. Among them, ACTs include tumor-infiltrating lymphocyte (TIL) therapy, engineered TCR therapy, CAR T therapy, and natural killer cell (NK) therapy (Jiang et al., 2019; Sukari et al., 2019; Titov et al., 2021). The main treatment steps for ACTs are as follows: First, T cells from patients are collected for ACT treatment, and then the cells are genetically engineered to produce chimeric antigen receptors on their surfaces. The cells are copied for two to three weeks until enough cells are injected into the patient’s body’s bloodstream (Dudley et al., 2010; Besser et al., 2013).

MAbs, as effective antitumor tools, have significantly advanced cancer therapy because of their targeting specificity and virtually no “off-target” effects compared to most small molecule drugs (Nasiri et al., 2018; Zhao et al., 2020b; Gu et al., 2021). In recent years, immunotherapy has been widely used in the clinical treatment of tumor diseases and has shown remarkable therapeutic potential (Gurunathan et al., 2021). Although immune checkpoint blockers have been shown to be effective against many types of advanced cancer, the overall patient response rate is still less than 30% (Carlino et al., 2021; Lommerts et al., 2021). This is mainly due to the immunomodulatory interactions between tumor cells and the ITM (Ma et al., 2022), which jointly mediate the immune tolerance of a tumor and, correspondingly, affect the positive response to immunotherapy (Kyu Shim et al., 2022). Thus, there is an urgent need to improve current cancer immunotherapies.

Cytokines are secreted by various of cell types and are regulators of cell activity (Konjevic et al., 2019). Cytokines are key signaling proteins in TME and have pleiotropic properties. In terms of tumor promotion, TGF-β, IL-1β, and CXCL12 can promote the survival and proliferation of tumor cells, TNF and IL-6 can cause the disorder of cytokine regulation and promote tumor inflammation, IL-10, IL-4, and TGF-β can be expressed, TNF, IL-6, and various chemokines can also promote angiogenesis (Zhou et al., 2021). Since the mixture of cytokines present in the tumor microenvironment shapes host immunity, therapeutic manipulation of the cytokine environment constitutes a strategy for stimulating protective responses.

Interferon-α, which produces anti-tumor effects in several hematological malignancies and solid tumors (Chen et al., 2019a). In malignant melanoma, randomized clinical trials have confirmed that interferon-α reduces the risk of recurrence after surgical resection of local lymph node metastases (Khammari et al., 2020). The mechanisms underlying the anti-tumor activity of IFN-α are not fully understood, but may include direct effects on tumor cells in addition to immune stimulation. Systemic administration of other cytokines may result in modest clinical benefit. Finally, although granulocyte colony-stimulating factor (G-CSF) and erythropoietin do not directly target tumors, they are widely used to improve the hematotoxicity of progressive cancer and cytotoxic therapies (Scharf et al., 2020). Systemic cytokine administration has achieved only modest therapeutic benefits, but the efficacy of cytokine therapy has been poorly, and many adverse reactions have resulted in intolerance in some patients, and the clinical progress on cytokine therapy has been limited.

## 3 Nanoparticles

With the development of nanotechnology, various nanoparticle structures have been used as carriers to deliver therapeutic drugs or imaging tags to the site of tumor growth (Chung et al., 2020; Raj et al., 2021; Ye et al., 2021). These nanomaterials can not only carry a wide range of molecular drugs but also stabilize their biological activity (Yang et al., 2019) and increase their solubility in biological fluids (Guerrini et al., 2018). Over the past decade, several nanoparticle-based compounds have been approved to deliver packaged or coupled therapeutics (Kumar et al., 2021; Wang et al., 2022b). Initially, these nano drug delivery vectors were used to prevent drug degradation and promote enrichment at the tumor site, thereby extending the half-life of the drug (Wang et al., 2021; Xia et al., 2021). The design of these first-generation nano drug delivery vectors was based on the enhanced permeability and retention (EPR) effects of peripheral blood vessels in tumor tissue (Jain et al., 2015; Edgar and Wang, 2017; Souri et al., 2022). In addition, the tumor is characterized by dysfunctional lymphatic drainage, which helps nanoparticles remain in the tumor long enough to allow local nanoparticles to decompose and drugs to be released near the tumor cells (Wang and Thanou, 2010; Ali et al., 2021). However, the EPR effects vary by tumor type due to differences in vascular anatomy and permeability (Fang et al., 2020). Therefore, EPR-dependent passive targeting is inefficient and often leads to unpredictable clinical outcomes, especially in the context of metastatic cancer (Jia et al., 2017). One of the major challenges in specifically targeting cancer and tumors is that defective cells are often very similar in character to the healthy tissue around them (Wang and Thanou, 2010). To differentiate these cells, researchers use chemical/physical methods in different surface modification of nanoparticle targeting ligands (Thomsen and Klok, 2021) [antibodies (Arslan et al., 2021), antibody fragments (Marques et al., 2020), proteins (Yoo et al., 2019), small molecules (Jahangirian et al., 2019), the aptamer (Fu and Xiang, 2020) and peptides (Gao et al., 2021a), etc.]. These ligands should have excessive expression of receptors on specific cancer cells but normal or minimum expression in the normal healthy cells (Wang et al., 2022c). At the same time, these ligand molecules should have a high affinity for their homologous receptors and the innate ability to induce receptor-mediated endocytosis.

In addition, the size, shape, surface charge, and material properties of nanoparticles play a key role in drug half-life and biological distribution (Huang et al., 2022; Souri et al., 2022). Among them, the size and surface properties are key to the biological fate of NPs, as these parameters prevent them from being absorbed by mononuclear phagocyte system (MPS) (Liu et al., 2022a; Khan et al., 2022; Mills et al., 2022). A high curvature (resulting in a small size, < 100 nm) and/or a hydrophilic surface (as opposed to the hydrophobic surface of conventional nanoparticles) are needed to reduce opsonization reactions and subsequent clearance by macrophages (Couvreur and Vauthier, 2006; Ren et al., 2022). For example, Yu Zhang et al. reported a supramolecular assembled programmable nanodrug (PIAN) as an *in situ* cancer vaccine to stimulate multiple steps of the immune activation process and induce a powerful antitumor immune response (Zhang et al., 2021b). Supramolecular components produce complex structures from simple modules and are suitable for the preparation of nanostructures to meet the multifunctional requirements of cancer immunotherapy (Huang et al., 2020). In this study, the authors selected a supramolecular host–guest interaction for the one-step preparation of PIAN. This optimized PIAN led to a succession of multiple steps after being injected into the body, starting with partial enrichment of tumor tissue after intravenous injection; PIAN dissociation and release of PPCD (which mediates tumor cell killing and antigen release) and CpG/PAMAM (which mediates antigen capture and transfer to tumor-draining lymph nodes) were then promoted by high levels of reactive oxygen in the tumor microenvironment (TME), leading to antigen-presenting cell activation, antigen presentation, and a strong antitumor immune response. Meanwhile, the study also found that PIAN in combination with anti-PD-L1 antibodies had a synergistic effect on tumor growth.

### 3.1 Biomimetic nanoparticles for tumor immunotherapy

Biomimetic nanoparticles are nanoparticles/microparticles extracted from living organisms and composed of endogenous membrane fragments (e.g., cell membrane, extracellular vesicles) and exogenous substances (Muller et al., 2016; Niculescu and Grumezescu, 2022). Among various nanocarriers, cell membranes are mainly derived from cancer cells, neutrophils, natural killer cells, macrophages, red blood cells, and platelets (Dash et al., 2020; Oroojalian et al., 2021; Lei et al., 2022). Hybrid membranes that combine the functions of multiple cell types also fall into this category due to the functional limitations of single cell types (Chen et al., 2020). In addition to the intact membrane, cells can also secrete exosomes and microvesicles. According to previous reports, the preparation of cell membrane materials with different cell types was as follows. First, the source cells were washed three times in Hank’s Balanced salt solution. Secondly, the cells were suspended in hypotonic lysis buffer and lysed by sonication and centrifugation. Eventually, the collected precipitate is cell membrane material (Liu et al., 2021b). These biological vectors inherit structural and functional complexity from the original donor and act as natural substances to reduce unnecessary immune responses and avoid direct elimination (Pan et al., 2021).

### 3.2 Bionic NDDS based on biomacromolecule

High-density lipoprotein (HDL) is a naturally occurring nanoparticle that is biocompatible, nonimmunogenic, and fully biodegradable (Ma et al., 2018; Iqbal et al., 2021). These endogenous particles can circulate over long periods of time and transport lipids and proteins from donor cells to recipient cells. Due to its inherent targeting properties, HDL is considered a promising DDS. Among them, apolipoprotein E (ApoE) is a polymorphic protein, including ApoE4, ApoE3, and ApoE2 (Marais et al., 2021). It is noteworthy that ApoE3 is the most dominant ApoE subtype found in healthy people and is used to construct HDL nanoparticles (Chernick et al., 2020). Recently, Wang et al. designed a tumor-penetrating lipoprotein that used naturally occurring HDL as a multifunctional nanoplatform for amplifying fluorescence imaging to guide the codon activation of ICG and small interfering RNA targeted HIF-1α (siHIF) in photogene therapy (Wang et al., 2022d). They designed a multimodal permeable HDL fused to the permeable peptide tLyP-1 to simultaneously improve the encapsulation efficiency of ICG and siRNA. The photosensitizer (ICG) was encapsulated in the hydrophobic core of the nanocarrier, and the cholesterol-conjugated siRNA (Chol-siHIF) was fully embedded in the phospholipid monolayer, prevented from enzymatic degradation, enabling the nanoparticles to be used for effective optogenetic therapy. The tLyP-1 peptide contains a C-terminal rule (CendR)-like sequence that binds to the NRP-1 receptor on tumor neovascular endothelial cells (Zhu et al., 2018), facilitating drug diffusion into extravascular tumors and penetration into deep tumors. In addition, HDL nanoparticles can not only effectively cross the blood-brain barrier and carry payloads to gliomas *in situ*, but also modulate the TME, thereby inhibiting tumor growth (Iqbal et al., 2021). Similarly, Zhou et al. (2020) developed a biomimetic nanoparticle modified with ApoE3 for efficient vaccine delivery to DC to enhance cancer immunotherapy by leveraging enhanced antigen presentation in DC for micropinocytosis. In 2021, Li et al. (2021c) demonstrated that phospholipid nanoparticles (PL1) efficiently delivered mRNA (CD137 or OX40) to T cells *in vitro* and *in vivo*. In multiple tumor models, the combination of PL1-OX40 mRNA and anti-OX40 antibody exhibited significantly increased antitumor activity compared to the anti-OX40 antibody alone. Moreover, this treatment significantly improved the immunotherapy effect of anti-PD-1 ^+^ anti-CTLA-4 antibodies. According to mechanism studies, PL1-OX40 mRNA can induce the activation of various immune cells, including T cells and dendritic cells (DCS). This study provided a platform for delivering costimulatory receptor mRNA to immune cells to enhance antitumor immunity.

### 3.3 NDDS based on cancer cells

**FIGURE 2:**
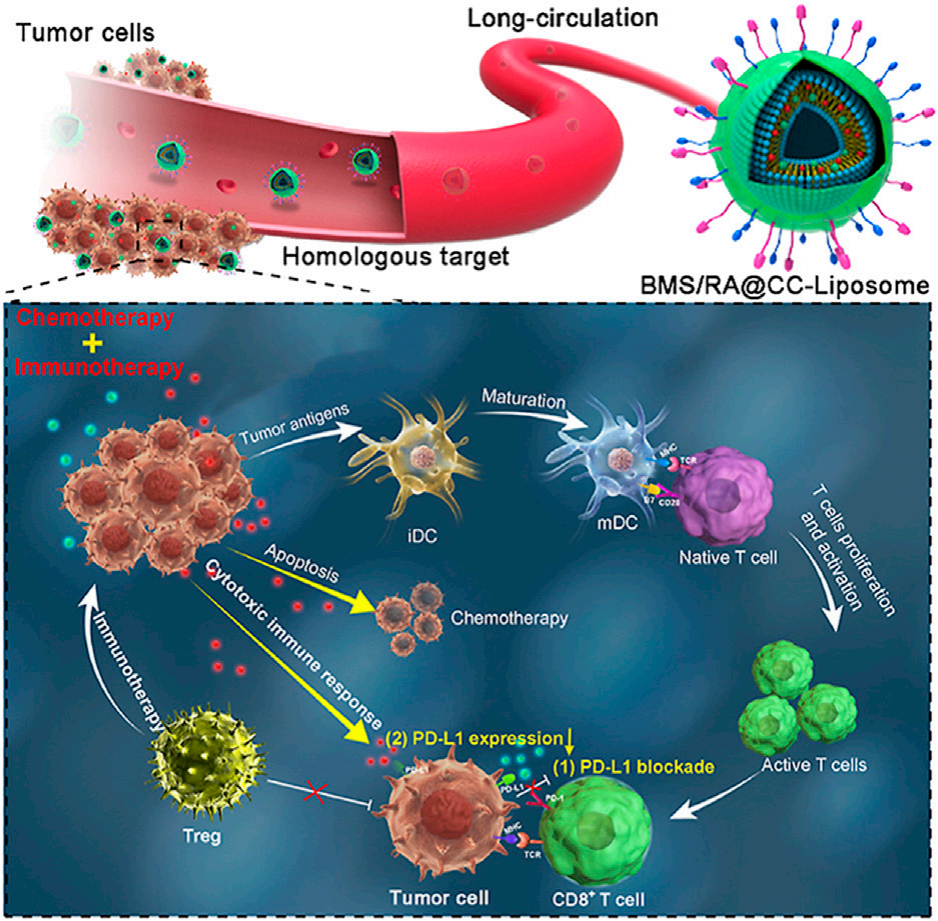
NDDS based on tumor membrane camouflage for hypoxic tumor chemotherapy and immunotherapy. Copyright Year 2022, Acta Pharmaceutica Sinica B (Yao et al., 2022).

Checkpoint block-based immunotherapy has shown unprecedented efficacy in cancer treatment, but its clinical implementation is limited by the low rate of host antitumor response (Jing et al., 2015). Recent studies have found that some chemotherapeutic drug-mediated tumor eradication can activate the immune system, and this enhanced immune response may further improve treatment efficiency through a synergistic mechanism (He et al., 2016; Luo et al., 2019). Yao et al. (2022) constructed a synergistic antitumor platform (designated BMS/RA@CC liposome) using CT26 cancer cell biomimetic nanoparticles combined with chemotherapeutic agents (RAV) and PD-1/PD-L1 blocking inhibitors (BMS-202) to significantly enhance antitumor immunity Figure 2. Meanwhile, this study mainly used cyclic peptide RA-V as a chemotherapeutic drug to directly kill tumor cells (Xu et al., 2021), and BMS-202 as an anti-PD drug to induce anti-tumor immune responses (Mikhail et al., 2022), and was co-encapsulated in a pH-sensitive nanosystems. In addition, the BMS/RA@CC liposomes could selectively target CT26 cells by taking advantage of the homologous adhesion inherent in tumor cells. These experiments showed that BMS/RA@CC liposomes achieved an immune response induced by the PD-1/PD-L1 blockade, PD-L1 downregulation, and apoptosis induced by Ra-V. The system combines the film camouflage effect of tumor cells and takes advantage of chemotherapy and checkpoint blockade immunotherapy to systematically create an immunogenic TME and improve the therapeutic effect on hypoxic tumor cells.

### 3.4 Bionic NDDS based on immune cells

**FIGURE 3:**
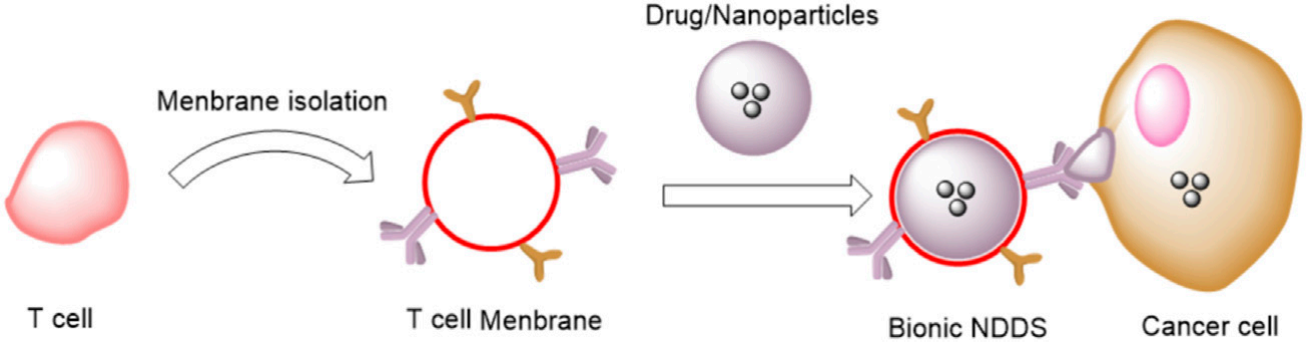
NDDS based on T cell membrane camouflage for immunotherapy.

Immune responses depend on communication and recognition between immune cells and between immune cells and other cells (Tavasolian et al., 2021). Immune cell recognition is based on their cell surface markers (Li et al., 2019). Due to these specific surface markers, immune cell membranes have unique functions and play special roles in adjuvant drug delivery, especially in tumor cell recognition and anti-tumor immunity Figure 3. For example, Chen et al. (2021) reported a tumor-associated macrophage membrane (TAMM) derived from primary tumors with a unique antigen homing affinity and immune compatibility. NaYF4:Yb, Er@NaYF4 conjugated with Rose Bengal (NPR) @TAMM (NPR@TAMM) could enhance the biocompatibility of nanoparticles and avoid the elimination of nanoparticles from the reticuloendothelial system. In addition, TAMM has a homing effect and immune compatibility, which can specifically drive the accumulation of nanoparticles in TME and competitively inhibit the interaction between TMA and tumor cells, thereby inhibiting tumor growth and metastasis (Hou et al., 2022). These advantages make TAMM very suitable as a potential immunotherapeutic agent, and its clinical efficacy will be more prominent when used in combination with other types of treatments such as PDT. The NPR@TAMM and near-infrared (NIR) groups showed an effective inhibitory effect on the growth of untreated distant tumors, suggesting that the NPR@TAMM treatment enhanced immunotherapy by eliciting a consistent distant effect. NPR@TAMM without the NIR group had a slight remote effect because PDT did not induce ICD. Furthermore, Deng et al. (2018) found that NK cell membranes could induce tumor-specific immune responses by targeting cancer cells and inducing polarization of M1 macrophages. They extruded the extracted NK cell membrane onto polymer nanoparticles loaded with the photosensitizer 4,4′,4′′,4′′′-(porphine-5,10,15,20-tetrayl) tetrakis (benzoic acid) (TCPP). The NK cell membranes enable NK-NPs to induce proinflammatory M1-macrophage polarization in tumors, leading to cell membrane immunotherapy. Nk-NPs can induce damage-associated molecular patterns (CRT exposure, ATP secretion, and HMGB1 release) in dying tumor cells through PDT-induced ICD, thereby enhancing the effect of NK cell membrane immunotherapy. Specifically, immunogenic PDT enhances NK cell membrane immunotherapy and significantly improves the infiltration of the effector T cells (CD4^+^ T cells and CD8^+^ T cells) in tumors, thereby effectively inhibiting both primary and distal tumors.

In order to overcome the limitations of current cancer treatments, Kang et al. prepared biomimetic nanoparticles using poly (lactic-co-glycolic) acid (PLGA) NPs coated on the T membrane of the EL4 cell line (TCMNP) (Kang et al., 2020). Its advantages lie in the fact that the EL4 cell line expresses various plasma membrane proteins as stably as the primary T cells, and TCMNPs can exert antitumor functions through proteins loaded in the core of PLGA nanoparticles and anticancer drugs. Similar to cytotoxic T cells, TCMNPs primarily target tumors through T cell membrane-derived proteins, killing cancer cells by releasing anticancer molecules and inducing Fas ligand-mediated apoptosis. Differently, TCMNPs can also eliminate TGF-β1 and PD-L1 on tumor cells by removing TGF-β1 and PD-L1. The results of Kang et al. showed that TCMNPs not only had a higher therapeutic effect than immune checkpoint blockade in melanoma but also showed a good antitumor effect in lung cancer.

### 3.5 Bionic NDDS based on platelets

**FIGURE 4:**
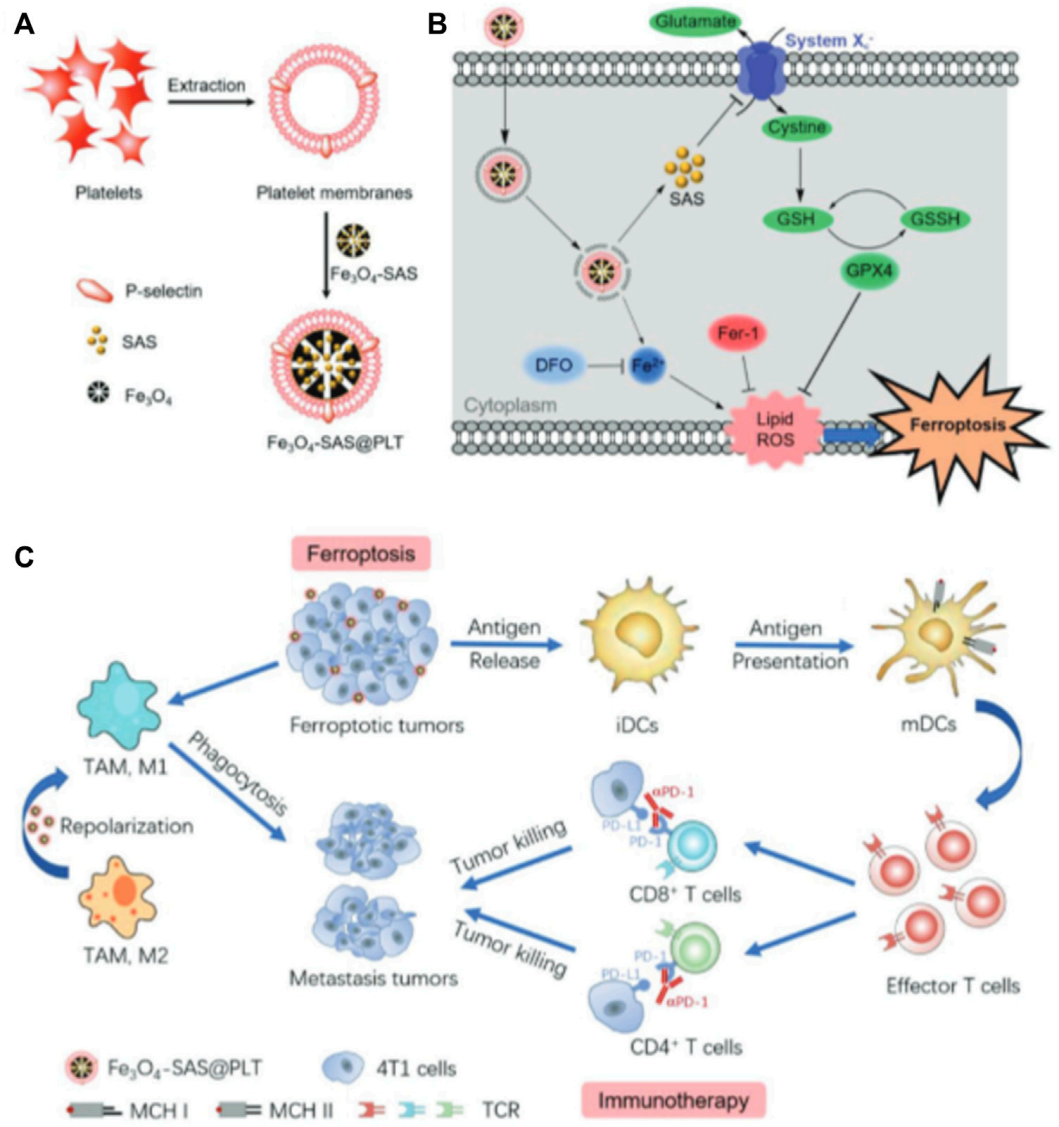
Magnetic nanoparticles coated with platelets for immunotherapy of iron-enriched cancer. Copyright Year 2020, Small (Jiang et al., 2020).

Platelets (PLT) are circulatory guards in the blood stream and have chemotactic effects on damaged vasculature and tumor tissue. Recent studies have shown that PLT membranes can be used as biomimetic coatings Figure 4, (Jiang et al., 2020; Dziendzikowska et al., 2021). Plasma membranes derived from human platelets have a variety of proteins, glycoproteins, and lipids and possess platelet-like properties, such as the ability to avoid macrophage recognition, capture of circulating tumor cells, and homing to inflammatory sites (Dziendzikowska et al., 2021). Bahmani et al. reported the development of a PLTs-membrane coated nanoparticle (PNP) specifically designed for intratumoral delivery of resiquimod (R848) to treat solid tumors (Bahmani et al., 2021). R848 acts as an agonist of TLR7 to stimulate dendritic cell activation and subsequent T cell initiation, leading to tumor-specific T cell immune response and immunity. Studies have shown that PNP loaded with R848 (PNP-R848) remains longer at the tumor site and improves cell interactions in the TME. This enables the drug to exhibit a significant biological activity when administered intratumorally, even at a low dose of R848, which would otherwise be ineffective for systemic administration. In this study, the authors found that biomimetic PLT-derived membranes can effectively increase the interaction of PNP-R848 with various cells in the TME and improve the bioavailability of R848. Therefore, this platelet-based biomimetic drug delivery system can significantly reduce the required dose of R848 while maintaining its therapeutic potential.

Meanwhile, Jiang et al. found that loading sulfasalazine (SAS) into magnetic nanoparticles (Fe_3_O_4_) and masking it with a platelet membrane (Fe_3_O_4_-SAS@PLT) could improve the treatment of tumors (Jiang et al., 2020). The platelet membrane coating imparts the ability of Fe_3_O_4_-SAS@PLT to evade and target tumor metastasis. Fe_3_O_4_-SAS@PLT may accumulate at the tumor site after intravenous injection. Iron Fe_3_O_4_@PLT mediated iron death induced an effective immune response that significantly promoted PD-1 blocking therapy in a mouse model of metastatic 4T1 tumors. Meanwhile, proteomic analysis was performed to elucidate the mechanism by which Fe_3_O_4_-SAS@PLT mediated iron death enhanced the antitumor immune response. This is a biomimetic magnetic nanoplatform (Fe_3_O_4_-SAS@PLT) to enhance effective iron death sensitivity and improve cancer immunotherapy. In addition, it has been found that Fe_3_O_4_-SAS@PLT could not only effectively trigger the iron death of tumor cells by inhibiting the X_c_^−^ transport pathway of the glutamate–cystine reverse transport system (Yuan et al., 2021) but could also induce an effective immune response and enhance the therapeutic effect of PD. The combination of Fe_3_O_4_-SAS@PLT-mediated iron death with immunotherapy effectively inhibited the growth of metastatic tumors compared to monotherapy. In addition, proteomic analysis showed that Fe_3_O_4_-SAS@PLT-mediated iron death could repolarize macrophages from immunosuppressive M2 phenotype to antitumor M1 phenotype. In conclusion, iron death mediated by Fe_3_O_4_-SAS@PLT can enhance systemic antitumor immunity along with the repolarization of tumor-associated macrophages, providing a new approach for clinically applicable collaborative immunotherapy of cancer. Due to the excellent biocompatibility and biosafety of Fe_3_O_4_-SAS@PLT nanoparticles, the main component in the design, this treatment approach is expected to play a role in the fight against cancer.

### 3.6 Bionic NDDS based on erythrocyte membrane

The erythrocyte membrane (EM) is highly biocompatible and has native functional proteins of the original cell (Gao et al., 2017). Currently, EM has been used to decorate nanoparticles to hide them for long periods of time, extending the half-life of nanoparticles in the body (Liu et al., 2021c). For example, Liang et al. (2019) prepared a bionic black phosphorus quantum dots (BPQDs) preparation for *in situ* induction of apoptosis in triple negative breast cancer cells. Meanwhile, a near-infrared (NIR) laser was used to stimulate the immune system to eliminate residual and metastatic cancer cells.

EM has been investigated as a suitable drug carrier for cancer treatment due to its essential biocompatibility and non-immunogenicity (Javed et al., 2021; Srivastava et al., 2022). At the same time, proteins, polysaccharides, and other rich “self-markers” play a key role in suppressing immune attack (Qin et al., 2020). However, single EM coated nanoparticles only have a long cycle time and lack active tumor targeting ability. According to the findings, cancer cell membrane (CM)-coated nanoparticles can improve the ability to target tumor tissues through the homologous binding ability of intercellular tumor and membrane proteins (Fan et al., 2021). Therefore, EM/CM hybrid membrane-coated nanoparticles have both immune camouflage ability and tumor targeting ability. Xiong et al. (2021) designed a hybrid membrane (IEM) composed of a mouse derived ID8 ovarian cancer cell membrane (ID8-CM) and CM. Then, IEM was coated on indocyanine green (ICG)-loaded magnetic nanoparticles (Fe_3_O_4_-ICG@IRM) for synergistic photothermal immunotherapy of ovarian cancer. Due to the basic properties of the CD47 protein on red blood cells, these carefully synthesized nanoparticles are different from bare Fe_3_O_4_-ICG NPs or ID8-CM-coated NPs (Fe_3_O_4_-ICG@ID8-CM), can significantly prolong the cycle time, and because cancer cells express surface adhesion molecules with homologous adhesion domains, they can actively target ID8 tumors. In addition, tumor antigens on IRM can also induce an antitumor immune response when Fe_3_O_4_-ICG@IRM is phagocytized by the spleen or lymph nodes. Meanwhile, Fe_3_O_4_-ICG@IRM NPs not only showed excellent PTT and an *in vivo* tumor temperature close to 60°C, but they could also further release whole cell tumor antigen through hyperthermo-induced tumor necrosis, enhancing antitumor immunotherapy. Similarly, Wang et al. (2020b) designed a novel pH-responsive copolymer micelle (dextran grafted poly (histiacid) copolymer) disguised as a CM/EM hybrid membrane to be prepared for the targeted delivery of selective CSF-1R inhibitors: BLZ-945 (abbreviated DH@ECM) to TAMs to consume TAMs. The prepared DH@ECM had good particle size (∼190 nm) and good immune camouflage and tumor homology targeting properties when injected intravenously into the blood system. DH@ECM had pH response characteristics and a unique “membrane escape effect” in the acidic TME, and promoted TAMs recognition and internalization through glucan-CD206 receptor-specific interactions. The experimental results indicate the therapeutic potential of DH@ECM in tumor immunotherapy.

## 4 Conclusion

In recent years, immunotherapy has shown good clinical effects, but the low response rate and off-target side effects hinder its wide application. The latest progress made in nanotechnology is expected to improve the efficacy of cancer immunotherapy (Gao et al., 2021b). However, concerns about the efficiency of cancer nanodrugs, the complex TME, patient heterogeneity, and systemic immunotoxicity have driven interest in developing more new methods (Landesman-Milo and Peer, 2016; Raza et al., 2021). Therefore, this paper summarized the latest progress of the biomimetic NDDS in improving immunotherapy and focused on the biomimetic nano technology derived from cell membranes and its application in cancer immunotherapy. The construction of BNDDS mainly uses membrane coatings from different cells to provide a simple method to introduce multiple functions into the same nanoparticles without complex synthesis technology. The bionic system includes EM, platelets, cancer cells, immune cells, and various microbes (Liu et al., 2022b). Membrane coatings provide unique properties such as increased blood circulation, RES escape, and the tumor-specific targeting of NPs (Oroojalian et al., 2021). Biomimetic NPs can enhance immune responses and easily target antigen-presenting cells, thereby effectively inhibiting tumor growth.

Although most reports emphasize the advantages of BNDDS, such DDS also have various limitations. For example, cell membrane coating technology still faces obstacles such as limited therapeutic effect in practical application, mainly due to the complex preparation process and relatively low production (Mitchell et al., 2021). Therefore, more research is needed to improve the performance of the biomimetic NDDS. This includes that in the process of scale-up production, uneven or incomplete coverage of the cell membrane on nanoparticles should be avoided to prevent unnecessary side effects in blood circulation (Sevencan et al., 2020). The repeatability of the manufacturing process and the preservation of functional surface proteins are critical, requiring additional monitoring and control measures (Zeng and Pu, 2020b). Therefore, more pioneering work is needed to address these issues in order to better understand how they work and pave the way for immunotherapy to develop more effective bionic DDSs with minimal side effects.

Currently, the rapid development of nanotechnology provides a new direction for improving the efficacy of clinical standard models. Cancer immunotherapy has shown efficacy in many cancer types (Chen et al., 2019b). However, only a few patients showed a positive response. To expand their applications, the use of BNDDS represents an attractive strategy to enhance the efficacy of immunotherapeutic agents by targeting and increasing their accumulation in tumor tissues. Recently, novel bio-inspired platforms have emerged to further improve nanomedicine delivery systems for cancer immunotherapy. Membrane coatings from cells of different origins provide a simple way to introduce multiple functionalities onto the same nanoparticle without complex synthesis techniques. As described here, various nanomaterials, including different biomimetic nanomaterials, play a unique role in improving the delivery of anti-cancer drugs. The simpler nanomaterials that can be developed, the greater their potential for clinical translation. Therefore, biomimetic nanoparticles with simple composition and mature production properties have the best potential to realize marketable nanodrugs.

BNDDS are biological vectors inspired by various cell membranes that inherit intrinsic properties of the source cells, including immune escape and tumor tropism (Luo et al., 2021). Theoretically, these vectors could be biocompatible with targeted delivery, long duration of action, and appropriate low side effects with diversified functional modifications. Because carriers are complex entities produced by living organisms, the transition of BNDDS from laboratory to clinical applications is hindered by multiple obstacles and synthesis. Most BNDDS are currently in the laboratory stage due to various technical and practical limitations (Fang et al., 2018; Chen et al., 2019c; Feng et al., 2019). Clinical applications of these drug delivery systems mainly include stability, volume production, safety and efficacy challenges (Madheswaran et al., 2019). One is the instability of BNDDS. The size, shape, composition, physical and chemical properties of nanoparticles vary with modifications and different sources, which can significantly affect their performance. In addition, long-term instabilities, as well as their difficulty in preservation and easy inactivation, hinder large-scale production of nanoparticles. Second, complex preparation methods make it difficult to achieve large-scale production, which limits the clinical transformation of BNDDS. More intelligent and efficient extraction and encapsulation methods are urgently needed to obtain biocompatible vectors with high purity, plasticity and repeatability. On one hand, manufacturing technologies are still in their infancy, involving immature separation, purification methods, low yields, improper modifications, and lack of efficiency in loading and delivering therapeutic payloads. Multiple purification methods such as filtration, hyper centrifugation and targeted ligand modification may adversely affect the quality and quantity of the finished product. In order to generate versatile nanoparticles, specific membrane decorations will inevitably be required, which may increase the risk of adverse side effects. For example, excess immune membrane-coated nanoparticles may promote inflammatory responses through interacting with the immune system, resulting in the release of pathogenic agents. Third, the biosecurity and bioactive effects of BNDDS in humans are still unclear. Translating these tissue-specific, non-toxic, non-immunogenic delivery technologies into clinical practice requires comprehensive *in-vivo* studies to estimate possible side effects and therapeutic effects. Therefore, the local and systemic toxicity of nanosystems to normal organs and tissues should be carefully studied and evaluated. However, despite these challenges, researchers continue to advance the design of BNDDS and expand their applications.
